# Supplementary material for: Simultaneous Determination of One-Carbon Folate Metabolites and One-Carbon-Related Amino Acids in Biological Samples Using a UHPLC–MS/MS Method
Source: Int J Mol Sci. 2024 Mar 19;25(6):3458. doi: 10.3390/ijms25063458 (PMC10971085; doi:10.3390/ijms25063458)
Supplement: Supplementary file 1 [file ijms-25-03458-s001.zip › Supplementary Table S1.pdf]

Supplementary Table S1. Some of the folate species measured in the literatures.

Folate species	Sample source	literatures
**5-CHOTHF**	**human serum**	**[39]**
**5-CH_3THF**		
**FA**		
**5-CH_3THF**	**human plasma**	**[40]**
**5-CH_3THF**	**human serum**	**[41]**
**FA**		
**Hcy**		
**5-CH_3THF**	**human serum**	**[42]**
**THF**		
**5fTHF**		
**510-CH+THF**		
**4-α-hydroxy-5-methyltetrahydrofolate(MeFox)**		
**5-CH_3THF**	**human plasma**	**[43]**
**DHF**		
**THF**		
**FA**		
**5-CH_3THF**	**mouse bone marrow cells and plasma, human whole blood, mouse muscle, liver, heart and brain samples**	**[44]**
**DHF**		
**THF**		
**FA**		
**510-CH+THF**		
**5-CHOTHF**		
