# Supplementary material for: Simultaneous Determination of One-Carbon Folate Metabolites and One-Carbon-Related Amino Acids in Biological Samples Using a UHPLC–MS/MS Method
Source: Int J Mol Sci. 2024 Mar 19;25(6):3458. doi: 10.3390/ijms25063458 (PMC10971085; doi:10.3390/ijms25063458)
Supplement: Supplementary file 1 [file ijms-25-03458-s001.zip › Supplementary Table S2.pdf]

Supplementary Table S2. Detection and quantitation limits of folate metabolites in the literature.

Folate	Sample source	LOD(nmol/L)	LLOQ(nmol/L)	literatures
5-CH_3THF	human plasma	1.51	4.51	[39]
FA	whole blood	-	2.27	[40]
THF		0.23	1.12	
510-CH+THF		0.22	1.10	
5-CHOTHF		0.32	1.06	
5-CH_3THF		0.22	1.09	
DHF		-	-	
5-CH_3THF	plasma	6.00	8.00	
Hcy		0.50	1.00	[45]
Cys		0.50	1.00	
Met		2.00	3.00	
5-CH_3THF	plasma	2.20	6.30	[46]
5-CH_3THF	plasma	1.00	3.00	[31]
FA		1.00	3.00	
THF		0.99	2.30	
510-CH+THF		3.01	15.00	
