# Supplementary material for: Simultaneous Determination of One-Carbon Folate Metabolites and One-Carbon-Related Amino Acids in Biological Samples Using a UHPLC–MS/MS Method
Source: Int J Mol Sci. 2024 Mar 19;25(6):3458. doi: 10.3390/ijms25063458 (PMC10971085; doi:10.3390/ijms25063458)
Supplement: Supplementary file 1 [file ijms-25-03458-s001.zip › Supplementary Table S3.pdf]

Supplementary Table S3. Folate standards used in this study.

	Standard	Abbreviated name	Formula	
1	(6R,S)-5-methyl-5,6,7,8-tetrahydrofolic acid, calcium salt	5-CH_3-Glu.Ca	$C_{20}H_{25}N_7O_6$.Ca	Schircks Laboratories
2	Folic acid	FA	$C_{19}H_{19}N_7O_6$	MYM biological technology company
3	(6R,S)-5,10-methenyl-5,6,7,8-tetrahydrofolic acid chloride	5,10-CH^+THF.Cl	$C_{20}H_{22}N_7O_6$.Cl	Schircks Laboratories
4	10-formylfolic acid	10-CHOFA	$C_{20}H_{19}N_7O_7$	Schircks Laboratories
5	(6R,S)-5-formyl-5,6,7,8-tetrahydrofolic acid, calcium salt	5-CHOTHF.Ca	$C_{20}H_{23}N_7O_7$	Schircks Laboratories
6	(6S)-5,6,7,8-tetrahydrofolic acid	THF	$C_{19}H_{23}N_7O_6$	Schircks Laboratories
7	*P*-Aminobenzoyl-L-glutamic acid	pABG		Schircks Laboratories
8	DL-homocysteine	HCY	$C_4H_9NO_2S$	Sigma
9	S-adenosyl-L-methionine	SAM	$C_{15}H_{22}N_6O_5S$	Sigma
10	L-Methionine	Met	$C_5H_{11}NO_2S$	Sigma
11	S-(5′-Adenosyl)-L-homocysteine	SAH	$C_{14}H_{20}N_6O_5S$	Sigma
12	L-Theanine	Thea	$C_7H_{14}N_2O_3$	Sigma
13	Methotrexate	MTX	$C_{20}H_{22}N_8O_5$	Ark Pharm
